# Trauma care during the COVID-19 pandemic in the Netherlands: a level 1 trauma multicenter cohort study

**DOI:** 10.1186/s13049-021-00942-x

**Published:** 2021-09-08

**Authors:** Nadia A. G. Hakkenbrak, Sverre A. I. Loggers, Eva Lubbers, Jarik de Geus, Stefan F. van Wonderen, Eva Berkeveld, Sarah Mikdad, Georgios F. Giannakopoulos, Kees J. Ponsen, Frank W. Bloemers, COVID-trauma collaborator group, Lore van Riel, Erik Bakkum, Gulsum Z. Nasim, Anneke van den Brink

**Affiliations:** 1 Department of Trauma Surgery, Amsterdam UMC, Room 7F-002, De Boelelaan 1117, 1081 HV Amsterdam, The Netherlands; 2 Trauma Unit, Department of Surgery, Northwest Clinics, Alkmaar, The Netherlands

**Keywords:** COVID-19, Trauma burden, Emergency department, Injury

## Abstract

**Purpose:**

The coronavirus (COVID-19) pandemic has caused major healthcare challenges worldwide resulting in an exponential increase in the need for hospital- and intensive care support for COVID-19 patients. As a result, surgical care was restricted to urgent cases of surgery. However, the care for trauma patients is not suitable for reduction or delayed treatment. The influence of the pandemic on the burden of disease of trauma care remains to be elucidated.

**Methods:**

All patients with traumatic injuries that were presented to the emergency departments (ED) of the Amsterdam University Medical Center, Location Academic Medical Center (AMC) and VU medical center (VUMC) and the Northwest Clinics (NWC) between March 10, 2019 and May 10, 2019 (non-COVID) and March 10, 2020 and May 10, 2020 (COVID-19 period) were included. The primary outcome was the difference in ED admissions for trauma patients between the non-COVID and COVID-19 study period. Additionally, patient- and injury characteristics, health care consumption, and 30-day mortality were evaluated.

**Results:**

A 37% reduction of ED admissions for trauma patients was seen during the COVID-19 pandemic (non-COVID n = 2423 and COVID cohort n = 1531). Hospital admission was reduced by 1.6 trauma patients per day. Fewer patients sustained car- and sports-related injuries. Injuries after high energetic trauma were more severe in the COVID-19 period (Injury Severity Score 17.3 vs. 12.0, p = 0.006). Relatively more patients were treated operatively (21.4% vs. 16.6%, p < 0.001) during the COVID-19 period. Upper-(17.6 vs. 12.5%, p = 0.002) and lower extremity injuries (30.7 vs. 23.0%, p = 0.002) mainly accounted for this difference. The 30-day mortality rate was higher during the pandemic (1.0 vs. 2.3%, p = 0.001).

**Conclusion:**

The burden of disease and healthcare consumption of trauma patients remained high during the COVID-19 pandemic. Results of this study can be used to optimize the use of hospital capacity and anticipate health care planning in future outbreaks.

## Introduction

During the past year, the novel severe acute respiratory syndrome coronavirus 2 (COVID-19) has caused a pandemic resulting in severe healthcare challenges and socioeconomic consequences worldwide. COVID-19 forced the current healthcare system to adapt promptly to the increasing demand for respiratory- and intensive care support. The number of COVID-19 infected patients increased rapidly from the beginning of March 2020 in the Netherlands. Major adaptation in healthcare allocation across all hospital departments was required due to the nationwide rising number of COVID-19 infections and exponential growth in hospital- and intensive care unit (ICU) admissions. The Dutch government implemented national measures to limit the spread of the virus amongst the Dutch population. Ultimately, leading to a national “intelligent lockdown” in March in order to prevent flooding of the national health care system [1]. Besides the existing hygiene measures, social distancing, quarantine in case of symptoms and advice to work from home as much as possible, additional compulsory measures by the government were implemented: closing all retail shops, restaurants, bars, sports- and fitness clubs, schools, universities, and childcare facilities, prohibiting events, nursing home visits and restricting in- and outdoor groups to a maximum of three persons or household. No curfew was implemented.

The number of daily positive polymerase chain reaction (PCR) tests for COVID-19 peaked on the 10th of April and the excess mortality rate increased up to 2.300 deaths per week [2]. As a result of the increased hospital- and ICU-requirement for COVID-19 patients, planned surgical procedures were cancelled and operating theatre time was reserved for urgent cases of surgery. However, not all surgical care (such as the care for trauma patients) is suitable for reduction or can be scheduled for delayed treatment. The urge for trauma care will continue to exist, due to simple falls, domestic accidents, and violence and needs to be anticipated upon.

Reports and experiences from previous pandemics, such as the SARS pandemic in 2003 demonstrated a substantial reduction in emergency department (ED) admissions during the peak of the pandemic [3, 4]. Moreover, recent studies regarding ED admissions in Europe during the COVID-19 pandemic reported similar results [1, 5, 6].

To date, it remains unclear to what extent the pandemic and national implemented measures have influenced the number and management of patients with traumatic injuries during the first wave of the COVID-19 pandemic in the Netherlands. Furthermore, the clinical characteristics and injury patterns of these patients remain to be elucidated, especially, since trauma patients make up a major part of patients in need of ED admission. Understanding the burden of disease of trauma patients during a pandemic is vital in order to anticipate and therefore optimize the use of hospital capacity, health care planning in future outbreaks and ultimately improve outcome for trauma patients during pandemics. It was hypothesized that the ED admission rate might decrease due to the implemented measures, but the need for admission and surgical intervention for trauma patients would decrease to a lesser extent. The aim of this study was to evaluate the burden of disease and characteristics of trauma patients in our regional trauma network, in the Northwest of the Netherlands, during the COVID-19 pandemic compared to a non-pandemic cohort.

## Methods

This retrospective cohort study was performed at the three leading hospitals of trauma care in the regional trauma Network of the province of North-Holland in the Northwest of the Netherlands. The regional trauma network (Dutch trauma registry of the National Network for Acute Care and SpoedZorgNet Amsterdam Medical Center) includes the Amsterdam University Medical Center (AUMC) location Amsterdam Medical Center (AMC) and location VU medical center (VUMC) and the teaching hospital Northwest Clinics in Alkmaar (NWC). These three hospitals are the three major trauma centers in the region, responsible for over 3 million inhabitants. The overall hospital bed capacity is set at 824 for the NWC (16 intensive- and medium care beds), 733 for the VUMC (36 intensive- and medium care beds) and 1002 for the AMC (50 intensive- and medium care beds).

### Study design

All patients with traumatic injuries admitted to the ED of the AUMC and the NWC during the two study periods between March 10, 2019, to May 10, 2019, (non-COVID) and March 10, 2020, to May 10, 2020, (COVID-19), were screened for eligibility. Patients treated by the department of emergency medicine, surgery, orthopedics, neurology or plastic surgery were assessed. Patients were included if they: sustained traumatic injuries (fractures, joint dislocations, ligamentous injuries, traumatic head-injuries, abdominal or thoracic injuries, vascular injuries, burns, deep lacerations (only those in need for surgical treatment in the operating theatre)), if they were admitted to the trauma ward after sustaining a high energy trauma (HET) or in case of admission for social indications (e.g., unable to return home, although no significant injuries were sustained). Patients were excluded in case of isolated head injuries without intracerebral injury treated by solely an emergency physician or neurologist, isolated contusions, abrasions, simple lacerations that did not required surgical intervention in the operating theatre, or if patients were transferred to another hospital, other than the participating trauma centers.

The two-month time frame (10th of March till 10th of May) was based on the timing of the additional measures implemented by the Dutch Government during the first peak wave of COVID-19 infections and liberalization of “lockdown” measures in May 2020.

Patient data was collected retrospectively from the electronic hospital charts. Data was collected and managed in Castor EDC according to a pre-defined datasheet and analyzed using the Statistical Package for the Social Sciences (SPSS) version 25 (SPSS, Chicago, III, USA). The results were reported according to the Strengthening the Reporting of Observational Studies in Epidemiology guidelines (STROBE) [7].

### Outcome measures

The primary outcome was the difference in the number of patients admitted to the ED with traumatic injuries during the non-COVID and COVID-19 study period. Secondary outcome measurements were patient characteristics (age, gender, medical history (e.g., psychiatric disease) and residential status), injury characteristics (trauma mechanism, place of injury, triage status, injury type, Injury Severity Score (ISS) and treatment), health care consumption and 30-day mortality rate. The triage status (T1-T5) was used to prioritize patients based upon urgency to receive medical care [8]. T1 is the most urgent category and concern patients in need of immediate medical care because of life threatening injuries, whereas T5 represents non-urgent care that theoretically could wait until the next day [8].

The injuries were divided into nine body regions; head, neck, face, thorax, abdomen, spine, upper extremities, lower extremities (including pelvic fractures) and external in accordance with the Abbreviate Injury Score (AIS) [9]. Injuries were scored as isolated injury or multiple injuries in case of polytrauma. Furthermore, for these nine body regions their consequent management (operative vs. non-operative) was registered.

In case of multiple injuries, the AIS was used to score the severity of the injured body regions ranging from 1 (minor injury e.g., contusion or simple fracture) up to 6 (untreatable injuries e.g., spinal cord transection above the level of C3, traumatic brain injury including brain stem laceration or massive bleeding) [9, 10].

Healthcare consumption was registered by recording the number of ED admissions, hospital admissions, length of hospital stay (LOS) (in days) and number of patients requiring outpatient follow-up.

### Statistical analysis

Statistical analysis was performed to assess for differences between the COVID-19 and non-COVID cohort. For descriptive analysis of continuous data, mean and standard deviation (SD) were reported. For categorical data, numbers and frequencies were reported. Comparison between the studied cohorts was done using Student’s T or Mann–Whitney U test (continuous data), or Chi-squared test or Fisher’s Exact test (categorical data). The P-value for statistical significance was set at p < 0.05. Data imputation was not used to correct for missing values. Data were analyzed using IBM® SPSS® Statistics version 24.0 (IBM, New York, NY, USA).

## Results

**Fig. 1:**
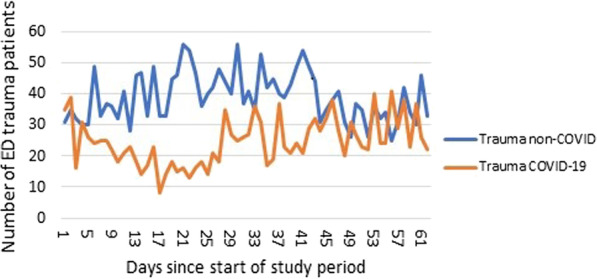
Daily number of patients admitted to the Emergency Department (ED) since the first day of the start of the national measures in the COVID-19 period compared to the non-COVID period during the same timeframe in 2019

A total of 3,954 trauma patients were admitted to the ED during the two study periods. A total of 1531 (39%) trauma patients were registered during the COVID-19 period and 2423 (61%) during the non-COVID period (Table 1). A decrease of 37% of trauma patients presented to the ED was found during the COVID-19 pandemic. A reduction of 46% was found in the AUMC (1090 non-COVID and 593 COVID-19) and 30% in the NWC (1333 non-COVID and 938 COVID-19) respectively. Prior to the pandemic, a daily average of 40 trauma patients were admitted to the ED, whereas 25 trauma patient ED admissions were registered during the first peak wave of COVID-19. Figure 1 shows the trend of ED admissions for trauma patients per day from the 10th of March till the 10th of May. The total number of patients across all specialties (not only trauma patients) admitted to the ED was 15,990 patients during the non-COVID period and 10,621 during the COVID-19 period. The daily number of ED admission decreased significantly during the COVID-19 period with an average of 257 patients during non-COVID to 171 patients per day during the COVID-19 period (p < 0.001). The proportion of trauma patients in relation to the total of ED admissions did not significantly decrease during the pandemic (15.1% during non-COVID and 14.4% during COVID, p = 0.231).

**Table 1:** Patient and mechanism of injury characteristics during the non-COVID and COVID-19 period

	Non-COVID (n = 2423)	COVID-19 (n = 1531)	*p* value
Age, yr (± std)	41.6 (± 26.8)	43.0 (± 27.2)	*0.100*
Age groups, n (%)			***0.014***
0–18	685 (28.3%)	397 (25.9%)	
19–39	556 (22.9%)	320 (20.9%)	
40–59	451 (18.6%)	309 (20.2%)	
60–79	464 (19.1%)	352 (23.0%)	
> 80	267 (11.0%)	153 (10.0%)	
Gender (female), *n* (%)	1122 (46.3)	722 (47.2%)	*0.312*
Medical History			
Cardiac, *n* (%)	148 (8.2%)	197 (9.7%)	*0.054*
Pulmonary *n* (%)	110 (4.6%)	149 (9.8%)	***0.000***
Diabetes, *n* (%)	115 (4.8%)	81 (5.3%)	*0.705*
Dementia, *n* (%)	45 (1.9%)	38 (2.5%)	*0.279*
Psychiatric, *n* (%)	89 (3.7%)	94 (6.2%)	***0.001***
Residential status			*0.438*
Community, *n* (%)	2333 (97.1%)	1483 (97%)	
Nursing home, *n* (%)	53 (2.2%)	35 (2.3%)	
Psychiatric clinic, *n* (%)	9 (0.4%)	6 (0.4%)	
Homeless, *n* (%)	2 (0.1%)	4 (0.3%)	
Rehabilitation center, *n* (%)	6 (0.2%)	1 (0.1%)	
Triage status			***0.002***
T1, *n* (%)	244 (6.1%)	124 (8.2%)	
T2, *n* (%)	278 (11.7%)	162 (10.7%)	
T3, *n* (%)	954 (40.3%	560 (37.0%)	
T4, *n* (%)	891 (37.6%)	571 (37.7%)	
T5, *n* (%)	103 (5.1%)	97 (6.4%)	
Multiple ED admission, *n* (%)	50 (2.1%)	65 (4.2%)	** < *0.001***
HET, *n* (%)	243 (10.1%)	177 (11.7%)	*0.067*
Setting of injury (public area), *n* (%)	1521 (66.2%)	871 (58.4%)	** < *0.001***
Mechanism of injury			** < *0.001***
Assault/domestic violence, *n* (%)	56 (2.4%)	41 (2.7%)	
Self-inflicted, *n* (%)	35 (1.5%)	36 (2.4%)	
Simple fall, *n* (%)	1140 (48.3%)	821 (54.3%)	
Traffic related, *n* (%)	445 (18.8%)	340 (22.5%)	
Work related, *n* (%)	98 (4.1%)	79 (5.2%)	
Sports related, *n* (%)	588 (24.9%)	196 (13.0%)	
Type of injury			***0.002***
Blunt, *n* (%)	2307 (96.4%)	1432 (94.1%)	
Penetrating, *n* (%)	56 (2.3%)	68 (4.5%)	
Burn (including chemical), *n* (%)	15 (0.6%)	9 (0.6%)	
Blast, *n* (%)	3 (0.1%)	0 (0.0%)	
Other, *n* (%)	13 (0.5%)	12 (0.8%)	

*n (%)* number of patients and percentage of number during that period, *yr* year, *std* standard deviation, *ED* emergency department, *HET* high energy trauma

### Patient characteristics

Age and gender did not differ between the two cohorts with a mean of 41.6 years (SD 26.8) in the non-COVID cohort and 43.0 years (SD 27.2) in the COVID-19 cohort (Table 1). The number of patients with a psychiatric disorder was higher in the COVID-19 cohort (6.2% vs. 3.7%, p < 0.001).

Relatively more patients were admitted to the ED multiple times during the COVID-19 period (4.2% vs. 2.1%, p < 0.001). Statistically less patients sustained their injuries in public areas (58.4% vs. 66.2%, p < 0.001). More patients suffered from penetrating injuries (2.3 vs. 4.5%) and car-related injuries (7.7% vs. 13.0%) during the COVID-19 period. (Table 1). The number of sports-related injuries was almost halved (24.9% vs. 13.0%). The number of trauma team activations during the COVID-19 period was reduced by approximately one per day (n = 70) and the number of T1 triaged patients was reduced by almost 50%. Ten out of 1531 patients (0.7%) were tested positive for COVID-19 by PCR in the ED or during hospital stay.

### Admissions

During the COVID-19 period, 108 fewer trauma patients were admitted to the hospital, although the proportion of patients that were admitted to the hospital during the COVID-19 period was relatively higher (24.0% vs. 31.0%, p < 0.001). The LOS was not significantly shorter during the COVID-19 period (6.0 vs. 5.1 days, p = 0.054). A total of 3474 admission days during the non-COVID period and 2388 admission days during the COVID-19 period were found. There were 1.6 fewer trauma patient hospital admissions per day during the COVID-19 period across the three participating hospitals (9.6 admissions per day in the non-COVID and 7.8 admissions per day in the COVID-19 period).

### Injury characteristics and management

The number of patients that sustained multiple injuries remained relatively stable around 10% (Table 2). The ISS of patients sustaining a HET was higher during the COVID-19 period (12.0 vs. 17.3, p = 0.006). Significantly more polytrauma patients (ISS ≥ 16) were seen during the COVID-19 period (n = 40 vs. n = 45, p = 0.009). Although an overall decrease in the number of injuries was seen, relatively more patients suffered from injuries to the head, face, neck, abdomen, and skin (Table 2).

**Table 2:** Injuries and their respective management during the non-COVID and COVID-19 period

	Non-COVID (n = 2423)	COVID-19 (n = 1531)	p value
Number of injuries, *n* (%)			0.781
Isolated injuries)	2115(87.4%)	1324 (86.7%)	
Multiple injuries	234 (9.7%)	158 (10.3%)	
Admitted without injuries*	70 (2.9%)	45 (2.9%)	
ISS scores after HET	12.0	17.3	**0.006**
Polytrauma (ISS ≥ 16), n (%**)	40 (17.1%)	45 (28.5%)	**0.009**
Injury region and management			
Head, *n* (% of total)	174 (7.2%)	150 (9.8%)	**0.002**
Nonoperative, *n* (%)	163 (94.2%)	139 (93.9%)	0.552
Face, *n* (% of total)	120 (5.0%)	99 (6.5%)	**0.026**
Operative, *n* (%)	11 (9.2%)	20 (20.2%)	**0.016**
Neck, *n* (% of total)	13 (0.5%)	19 (1.2%)	**0.014**
Nonoperative, *n* (%)	11 (84.6%)	17 (89.5%)	0.542
Thorax, *n* (% of total)	152 (6.3%)	103 (6.7%)	0.307
Nonoperative, *n* (%)	141 (92.8%)	97 (94.2%)	0.431
Abdomen, *n* (% of total)	32 (1.3%)	33 (2.2%)	**0.031**
Conservative, *n* (%)	16 (50.0%)	23 (69.7%)	0.086
Spine, *n* (% of total)	88 (3.6%)	51 (3.3%)	0.342
Nonoperative, *n* (%)	80 (90.9%)	43(84.3%)	0.184
Upper extremity, *n* (% of total)	1188 (49.0%)	779 (50.9%)	0.267
Operative, *n* (%)	148 (12.5%)	138 (17.6%)	**0.002**
Lower extremity, *n* (% of total)	874 (36.1%)	512 (33.4%)	0.095
Operative, *n* (%)	201 (23.0%)	157 (30.7%)	**0.002**
Skin, *n* (% of total)	55 (2.3%)	18 (1.2%)	**0.008**
Operative, *n* (%)	5 (9.1%)	3 (17.6%)	0.281
Surgery			
Surgery performed, *n* (%)	402 (16.6%)	327 (21.4%)	** < *0.001***
Total number of surgeries, n	439	388	-
Multiple surgeries, *n* (%)	30 (2.3%)	36 (3.1%)	0.158
Days till surgery, days (± std)	3.7 (± 5.1)	2.6 (± 4.0)	**0.001**
Delayed surgery (> 48 h.), *n* (%)	153 (12.1%)	94 (8.5%)	**0.002**
Admission for injuries	582 (24.0%)	474 (31.0%)	** < *0.001***
Hospital stay, days	6.0 (± 8.5)	5.1 (± 7.0)	0.054
Outpatient follow-up, *n* (%)	1885 (79.1%)	1167 (78.0%)	0.221
Physical follow-up, *n* (%)	1854 (76.5%)	880 (57.5%)	** < *0.001***

*n (%)* number of patients and proportion of number in percentage, *n (% of total)* number of patients and percentage of injuries treated operatively or nonoperatively as a percentage of total, *hrs.* hours, *std* standard deviation

*Patients admitted to a ward after a HET of social indications without obvious injuries

**As a percentage of patients sustaining multiple injuries

A higher proportion of patients was treated surgically during the COVID-19 period (21.7% vs. 16.8%, p < 0.001) (Table 2). During the non-COVID period 7.2 surgeries per day were performed compared to 6.4 surgeries in the COVID-19 period. In total, only 51 fewer surgeries were performed within the 30-day follow-up during the COVID-19 period. Fewer patients underwent delayed surgery in the COVID-19 period (12.1 vs. 8.5%, p = 0.002). The time until surgery was reduced by 1.1 days in the COVID-19 period (3.7 during the non-COVID vs. 2.6 in the COVID-19 period, p = 0.001).

The proportion of patients that sustained an upper extremity injury (UEI) or lower extremity injury (LEI) remained the same. However, significantly more patients were treated operatively for their UEI during the COVID-19 period (12.5% vs. 17.6.%, p = 0.002). The same accounted for LEI, where 23.0% of the patients were treated surgically during the non-COVID period compared to 30.7% of the patients during the COVID period (p = 0.002). The operative rate for extremity injuries did not significantly differ between the participating hospitals.

#### Patient characteristics for operated UEI

Patients were significantly younger during the COVID-19 period (46.7 vs. 53.2 years, p = 0.016). The ISS did not differ (p = 0.075), nor did the proportion of HET (14.7% vs. 14.2%, p = 1.000) or type of injury (p = 0.911). There were no differences between age groups of patients treated operatively for UEI (p = 0.200) and patients aged under18 (p = 0.111) and over 80 (p = 0.198).

#### Patient characteristics for operated LEI

The age and ISS of patients who were treated surgically for LEI did not differ during the COVID-19 period (61.5 vs. 59.0 years with p = 0.345 and 18.8 vs. 14.4 with p = 0.261 respectively). The proportion of HET was the same (18.1% vs. 17.2%, p = 0.888). There were no differences between age groups of patients treated operatively for UEI (p = 0.245), or for patient aged under18 (p = 0.671) and over 80 (p = 0.414). The mechanism of injury did not differ between cohorts (p = 0.197).

### Outpatient follow-up

The same proportion of trauma patients received a form of outpatient clinical follow-up (Table 2). However, significantly less patients were scheduled for physical outpatient follow-up during the COVID-19 study period (76.5 vs. 57.5%, p < 0.001). Instead, a great proportion of physical follow-up was replaced by phone or video-calls during the COVID-19 period.

### Mortality

The 30-day mortality rate did significantly differ during the non-COVID and COVID-19 period (1.0% versus 2.3%, p = 0.001). Mortality during the index hospitalization was also higher in the COVID-19 period (1.9% vs. 4.8%, p = 0.008). During the pandemic, one patient died in the ED and 24 during admission. In the non-COVID period, seven patients died in the ED, thirteen during admission, and one post-discharge in the 30-day follow-up period. The presumed causes of death did not differ significantly between the two cohorts (p = 0.279).

## Discussion

This study was the first multicenter study conducted at level 1 trauma centers in the Netherlands that investigated the burden of disease of trauma patients during the first peak wave of the COVID-19 pandemic in relation to the normal standard of care. The burden of disease of trauma patients admitted to the ED decreased significantly by more than a third during the first COVID-19 peak wave. However, the health care consumption remained significant. The management of trauma patients changed in several aspects.

We found that, despite the “intelligent’’ lockdown, only 1.6 fewer patients were admitted to the ED per day. No curfews or orders to stay at home were implemented by the Dutch government. This only led to a shift in trauma mechanism and not in a decrease of behavior that would prevent the chance of traumatic injuries. This resulted in a continuing number of patients sustaining traumatic injuries such as domestic-, car- and traffic-related accidents. The number of sport-related injuries was almost halved after the implemented measures. This is in line with other recent COVID-19 studies worldwide, that show a strong decline in admissions for trauma patients up to 45%, less trauma team activations, less traffic accidents, sport-related injuries, and shorter LOS [11, 12].

However, despite the reduced number of ED admissions, the relative percentage of patients in need for hospital admission increased by 7.0%. There are several explanations for these findings. First, a slight increase in elderly patients sustaining traumatic injuries was seen, as they are more often in need for hospital admission due to decreased self-sufficiency as a result of immobility compared to younger patients. Secondly, the proportion of patients suffering a traffic related HET or self-inflicted injury was higher. This resulted in a higher admission rate, as they are more often severely injured.

Furthermore, relatively more patients were treated surgically. This was an unexpected and remarkable finding. This could partly be explained by the higher ISS scores in HET patients, the slight decrease in the group of patients under18 and changes in injury setting and mechanisms.

Also, significantly more patients were treated surgically for extremity injuries. This finding could not be explained by a clinically relevant difference in age or type of injury. However, it could be suggested that due to the uncertainty of the reduced availability of operation theatre time during the pandemic, the threshold to immediately schedule patients for surgery was lowered. This is also reflected by the decreased time to surgery during the COVID-19 pandemic. Especially, in patients who sustained fractures with an alternate option for nonoperative treatment. These patients normally would have been re-evaluated at the outpatient clinic after one week with repeated imaging and in case of progressive fracture dislocation secondarily planned for surgery. Hypothetically, these patients were more often immediately planned for surgery because of fear of unavailability of future surgical operating theatre time. However, this was not explored in this study and remains a hypothetical factor since fracture types were not registered in this dataset.

Finally, a higher proportion of patients with psychiatric disorders were seen. This is most likely related to the increase of self-inflicted injuries. Studies addressing previous pandemics also demonstrate an increase of psychiatric disease during and after pandemics such as Ebola and SARS [13, 14]. In relation to the COVID-19 pandemic several studies also report an increase of psychiatric symptoms for psychiatric patients in need for additional treatment [15].

### Limitations

Some limitations of this study should be noted in accordance with the retrospective character of the study. Due to the extent of this cohort and in relation to the main study outcome, fracture types and classifications were not registered in this database. Therefore, the increase in operated extremity injuries could not be further assessed or related to the fracture type. The reasons for this increase remain hypothetical but nevertheless are an interesting finding. Due to the change of physical outpatient follow up into audio- or video- based follow up, functional outcome could not be properly evaluated. Evaluation of the impact of the change in outpatient follow up will be interesting in the near future for potentially improving time management, reduce the need for patient to travel to the hospital, and reduce health care costs without compromising the high standard of care for trauma patients [16]. Furthermore, ICU admission, ventilatory support, and ICU LOS were not explored in this study, but the formal threshold for trauma-related ICU admission remained unchanged.

This study is the first comparative multicenter study to address the changes in burden of disease of trauma patients in major trauma center in the Netherlands during the COVID-19 pandemic. The results of this study emphasize the importance of understanding the burden of disease in order to optimize the use of hospital capacity and anticipate health care planning for trauma care in future outbreaks in order to provide high quality care for trauma patients during pandemics as the hospital capacity for non-pandemic care decreases.

## Conclusion

Despite governmental restrictive measures to control the COVID-19 pandemic the burden of trauma-related disease remained high during the first-peak wave of the COVID-19 pandemic. The characteristics and management changed in several aspects. The overall in-hospital healthcare consumption was only marginally reduced and the number of surgically treated patients relatively increased. More severely injured patients and a higher percentage of patients in need for hospital admission were observed. Higher percentages of patients were treated surgically for extremity injuries. Results of this study can be used to optimize the use of hospital capacity and anticipate health care planning in future outbreaks for trauma patients.

## Data Availability

The dataset generated during and/or analysed during the current study are not publicly available due to patient privacy but are available from the corresponding author on reasonable request.
